# Time Savings and Surgery Task Load Reduction in Open Intraperitoneal Onlay Mesh Fixation Procedure

**DOI:** 10.1155/2015/340246

**Published:** 2015-07-09

**Authors:** Sanjoy Roy, Jeffrey Hammond, Jessica Panish, Pullen Shnoda, Sandy Savidge, Mark Wilson

**Affiliations:** ^1^Global Health Economics and Market Access, Ethicon, Somerville, NJ, USA; ^2^Medical Affairs, Ethicon, Somerville, NJ, USA; ^3^Preclinical Research, Ethicon, Somerville, NJ, USA; ^4^College of Life and Environmental Sciences, University of Exeter, Exeter, UK

## Abstract

*Background.* This study assessed the reduction in surgeon stress associated with savings in procedure time for mechanical fixation of an intraperitoneal onlay mesh (IPOM) compared to a traditional suture fixation in open ventral hernia repair. *Study Design*. Nine general surgeons performed 36 open IPOM fixation procedures in porcine model. Each surgeon conducted two mechanical (using ETHICON SECURESTRAP^TM^ Open) and two suture fixation procedures. Fixation time was measured using a stopwatch, and related surgeon stress was assessed using the validated SURG-TLX questionnaire. T-tests were used to compare between-group differences, and a two-sided 95% confidence interval for the difference in stress levels was established using nonparametric methodology. *Results*. The mechanical fixation group demonstrated an 89.1% mean reduction in fixation time, as compared to the suture group (*p* < 0.00001). Surgeon stress scores measured using SURG-TLX were 55.5% lower in the mechanical compared to the suture fixation group (*p* < 0.001). Scores in five of the six sources of stress were significantly lower for mechanical fixation. *Conclusions*. Mechanical fixation with ETHICON SECURESTRAP^TM^ Open demonstrated a significant reduction in fixation time and surgeon stress, which may translate into improved operating efficiency, improved performance, improved surgeon quality of life, and reduced overall costs of the procedure.

## 1. Introduction

Surgery is a complex procedure, which is often conducted under high pressure and potentially hazardous environment [1–5]. It is well documented that stress during surgery is common and can negatively impact surgeon performance and patient safety [1–5].

Another major stressor is workload, a multifaceted factor, determined by the interaction of the task demands, the circumstances under which the task is performed, and the skills, behaviors, and perceptions of the individual [6]. Workload has various dimensions, such as mental demands, physical demands, temporal demands, task complexity, situational stress, and distractions, all of which can increase surgeon stress [6–8]. For example, procedures that are complex or longer in duration trigger elevated stress levels because they are more physically and mentally demanding [9]. Additionally, increased mental demands and distractions can increase workload and stress, with deleterious effects on surgical performance. Further, it has been shown that reducing procedure time can decrease patient postsurgery in-patient time and leads to fewer unplanned admissions and fewer complications [10]. Procter et al. showed that surgical operative duration is associated with increased infectious complication rates and length of hospital stay after adjustment for procedure and patient risk factors [11].

Moorthy et al. found that a significantly higher number of errors occurred during a simulated laparoscopic task when stressors (simple verbal mathematical task, increased operating theatre background noise, and time pressure) were present, with effect being most pronounced when all the stressors were applied in combination [12]. Strategies to manage and reduce stress have been shown to improve performance and translate to a reduced number of errors [2, 5, 13]. These factors are especially important for open surgical procedures, which have been reported to be complex and have high workload [14].

Importantly, studies have shown that decreasing the workload can improve surgical performance and safety [15]. New medical devices offer time savings for surgical procedure and have been shown to improve operating room efficiencies [10] and hospital costs; therefore they offer a potential strategy for reducing surgical workload.

*Open Ventral Hernia Repair*. One such potentially complex open surgical procedure is the open intraperitoneal onlay mesh (IPOM) procedure for ventral hernia repair. The open IPOM technique is a hernia repair procedure wherein a mesh is placed over the hernia defect intra-abdominally [16]. Hernia repair surgeries are among the most commonly performed procedures in the world. Although disproportionately less in number compared to inguinal hernias, ventral/incisional hernia surgeries are considered more involved in nature and pose a significant burden to the healthcare system.

An assessment from 2012 estimated approximately 350,000 ventral hernia repairs annually in the United States, a majority of which adopted an open approach [17]. One global estimate predicts ventral hernia surgeries to be increasing to about 800,000 by 2017 at an annual growth rate of about 3% [18]. About half of these surgeries were expected to be in the US, which would disproportionately account for about 70% of the over $780 million global ventral hernia procedure market by 2017 [18].

Despite it being one of the more common surgical procedures, readmissions after open incisional hernia repair has not been examined widely and has not been included in recent assessments of the outcomes of such procedures [19]. A recent prospective nationwide study conducted in Denmark of elective incisional hernia repairs suggested that 30-day readmissions occurred in 13% of cases and that open repairs were associated with worse early outcomes [20]. Another study found that the duration of surgery (odds ratio: 1.35; 95% confidence interval: 1.05 to 1.73) and the presence of prior superficial or deep surgical-site infection (odds ratio: 2.39; 95% confidence interval: 1.32 to 4.32) were predictors of 30-day readmission after open ventral hernia repair [21].

There is a need for new medical devices and techniques, which can lower the surgeon stress experienced during Open IPOM repair of ventral/incisional hernias by shortening the duration of the operation and decreasing workload demands. One of the potential areas for stress and time reduction is the mesh fixation method, which can be performed either by traditionally hand-suturing the mesh or by using mechanical fixation devices.

The aim of this study was to evaluate potential time savings and reduction in task load associated with mechanical fixation of IPOM mesh with the ETHICON SECURESTRAP^TM^ Open device compared to suture fixation. The study also represents the first such examination of surgeon stress as a metric for new device evaluation.

## 2. Methods

Nine general surgeons, who were experienced with IPOM procedures, performed thirty-six open IPOM mesh fixation procedures on five female Yorkshire pigs.

To simulate a surgical environment, animals were anesthetized as per approved Institutional Animal Care and Use Committee (IACUC) protocol and were maintained under anesthesia for all procedures. The animals were implanted with a commonly available hernia patch, an oval-shaped composite mesh (11 cm × 14 cm). For suture fixation, PROLENE (polypropylene) Suture, PDS (polydioxanone) Suture, VICRYL (polyglactin 910 Suture), or any alternative sutures were used as per surgeon preference. The ETHICON SECURESTRAP^TM^ Open absorbable strap fixation device was used for mechanical fixation of the mesh. Animals were euthanized at the completion of the final scheduled procedure.

Two longitudinal incisions, approximately 7 cm long, were made cranial and caudal to the umbilicus through the skin, subcutaneous tissues, linea alba, and peritoneum to expose the abdominal cavity using a standard technique. Both defects were used for the evaluation of suture and ETHICON SECURESTRAP^TM^ Open fixation procedures in an alternating fashion.

Each surgeon performed two SECURESTRAP Open and two suture-based mesh fixations. As blinding was not feasible, the order of fixation procedures was randomly altered to ensure reduction in the effect of learning curve. The study design and outcomes of interest were discussed with the surgeons prior to the procedures, without any mention of the hypotheses.

### 2.1. Suture Fixation Procedure

Multiple transabdominal stay sutures were placed around the border of the skirted mesh, as per surgeon preference. The mesh was inserted into the abdominal cavity and placed over the created incisional defect with an overlap of approximately 3–5 cm beyond the edge of the defect. A transabdominal suture passer could be used to grasp the sutures and exteriorized through the abdominal musculature in order to hold the mesh against the abdominal wall in an appropriate anatomic and physiologic location. The sutures were tied externally in routine fashion. Timing of the procedure started once the surgeon placed the first suture in the mesh, and ended when the surgeon indicated that the last knot was tied.

### 2.2. Mechanical Fixation Procedure

The mesh was inserted into the abdominal cavity and placed over the created incisional defect with an overlap of approximately 3–5 cm beyond the edge of the defect. The mesh was secured to the peritoneal layer and underlying musculature using ETHICON SECURESTRAP^TM^ Open per the Instructions for Use. The straps were placed through the skirted portion of the mesh as per standard of care for hernia mesh fixation method and/or surgeon preference. Timing started at the insertion of the mesh and ended at the last firing.

The duration of each procedure was monitored by a timer and recorded for subsequent analysis.

Surgeon stress and workload were assessed using a validated questionnaire, the “Surgery Task Load Index” (SURG-TLX) [6], which was based on an original measure created for pilots (the NASA-TLX) [22].

The six dimensions (source of workload) for the SURG-TLX are as follows:

1. Mental demands: how mentally fatiguing was the procedure?
2. Physical demands: how physically fatiguing was the procedure?
3. Temporal demands: how hurried or rushed was the pace of the procedure?
4. Task complexity: how complex was the procedure?
5. Situational stress: how anxious did you feel while performing the procedure?
6. Distractions: how distracting was the operating environment?

Following each fixation (hand suture or mechanical), surgeons were asked to rate their experience with the procedure by marking an X on a visual analogue scale, (anchored between “low” and “high”) for each of the six dimensions of task load included in the SURG-TLX questionnaire.

After completion of all procedures, surgeons were asked for their response to pairwise comparisons between each set of the dimensions in terms of their perceived importance and relevance with respect to the procedure being studied.

### 2.3. Statistical Analyses

The procedures were compared between the two groups, namely, suture fixation and mechanical fixation, with ETHICON SECURESTRAP^TM^ Open. As each surgeon performed each procedure on the same pig and the same sized defect, these observations were considered paired.

The difference in time was calculated for each paired observation. Paired sample *t*-tests were used to compare differences, and a two-sided 95% confidence interval for the difference in fixation time was established using nonparametric methodology.

Responses to the SURG-TLX questionnaire were scored following guidance from the developers [6]. The raw score for each dimension (from the visual analogue scale) was adjusted by the respective weights calculated based on surgeons' responses to the pairwise comparisons of the different dimensions. Subsequently, a score for each dimension and an overall SURG-TLX score (summing all the dimension scores) were calculated. The differences between SURG-TLX scores from each paired observation (suture versus mechanical) were calculated and paired sample *t*-tests were again used to compare between-group differences.

A *p* value of <0.05 was considered significant for all statistical analyses.

## 3. Results

Nine surgeons participated in the study and performed open intraperitoneal onlay mesh fixation in an* in vivo* preclinical porcine model. A total of 36 IPOM procedures were performed, with each surgeon conducting two mechanical and two suture fixation procedures.

Tables 1 and 2 present the observed fixation time in minutes and computed overall SURG-TLX scores measuring surgery task load related stress. These tables also compute the differences on each outcome in the paired observations between the mechanical and suture fixation groups. All duration and most surgeon stress assessments were more favorable for the ETHICON SECURESTRAP^TM^ Open group.

Similar trends exist in the weighted observed scores for each dimension of the SURG-TLX questionnaire (Table 3).  Table 4 presents the summary measures of central tendency.

**Figure 1:**
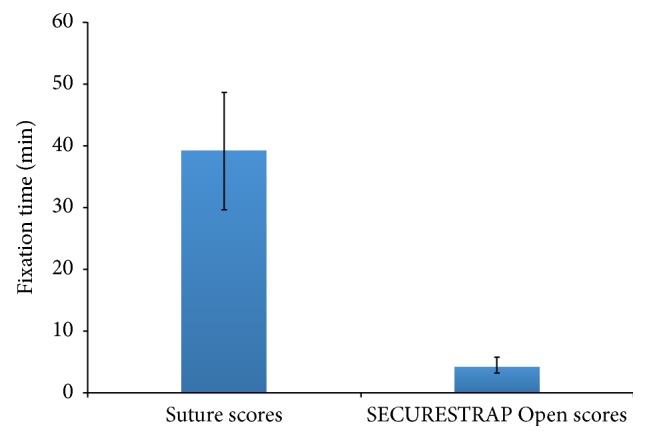
Time scores for suture and mechanical fixation groups.

Overall, the ETHICON SECURESTRAP^TM^ Open group demonstrated an 89.1% mean reduction in fixation time, as compared to the suture group (*p* < 0.00001). The mean fixation time in the suture group was 39.18 minutes (95% CI: 29.70–48.68), while the mean fixation time in the ETHICON SECURESTRAP^TM^ Open group was 4.27 minutes (95% CI: 3.20–5.83) (Figure 1).

**Figure 2:**
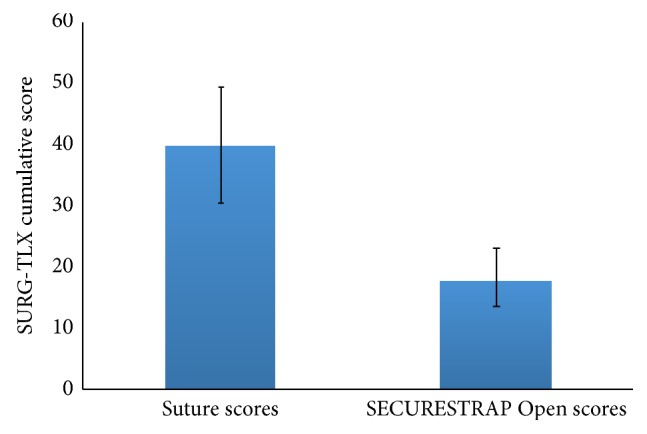
Task load (SURG-TLX) scores for suture and mechanical fixation groups.

Surgeon stress scores measured using SURG-TLX were 55.5% lower in the ETHICON SECURESTRAP^TM^ Open group, compared to the suture group (*p* < 0.001). The mean SURG-TLX scores were 39.94 (95% CI: 30.42–49.47) and 17.78 (95% CI: 12.62–22.93) in ETHICON SECURESTRAP^TM^ Open and sutures groups, respectively (Figure 2).

**Figure 3:**
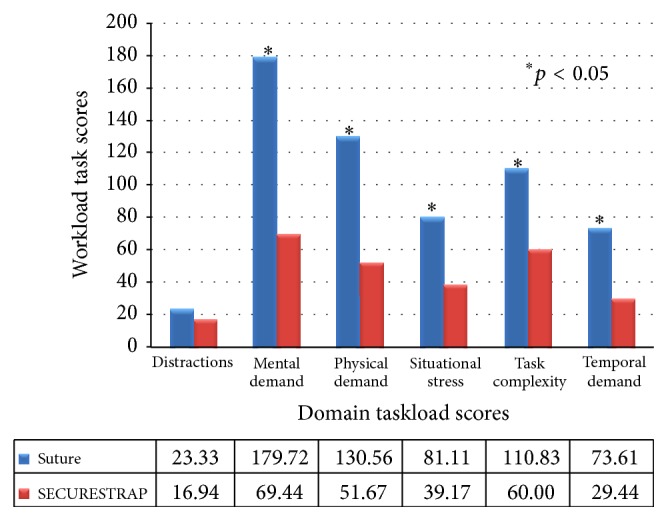
Task load (SURG-TLX) domain scores for suture and mechanical fixation groups.

Scores in five of the six dimensions of sources of stress in the SURG-TLX instrument, namely, mental demands, physical demands, temporal demands, task complexity, and situational stress, were significantly lower for fixation with ETHICON SECURESTRAP^TM^ Open compared to suture fixation (*p* < 0.05; Figure 3). The mean score for the remaining dimension (distractions) was also lower for the mechanical fixation group but did not reach statistical significance.

## 4. Discussion

Time pressure and increased workload are two common causes of surgeon stress, which has been shown to lower surgical performance and increase risk to patient safety [2, 3, 5]. An interesting theoretical framework for explaining individual differences in stress response in surgery is the biopsychosocial model (BPSM) of challenge and threat [23]. The framework is predicated upon the surgeon's first evaluation of the demands of a procedure compared against the possession or availability of necessary resources to cope effectively with such demands [23]. When resources are perceived to be sufficient, a “challenge” state occurs, resulting in a surgeon experiencing more favorable cognitive, affective, physiological, and behavioral outcomes [23–25]. In contrast, if a surgeon perceives that she/he does not possess the resources required to meet the demands of the situation, a “threat” state emerges [23]. Thus, poor surgical performance may arise when surgeons evaluate a stressful event as a “threat” instead of a “challenge” [26–28].

Research also suggests that interventions that help modify surgeons' evaluations of stressful events to ensure they are perceived as a “challenge” (as opposed to a “threat”) situation would improve surgical performance and patient care and could also have important cardiovascular health implications for surgeons experiencing chronic threat states [23]. Strategies to reduce procedure time and workload would as a result improve surgical performance and reduce errors and can potentially lower medical costs [4, 11, 29].

Our study evaluated the use of ETHICON SECURESTRAP^TM^ Open for mechanical fixation of IPOM mesh as compared to standard suture technique, in a preclinical model. We found an 89.1% reduction in fixation time and 55.5% lower surgeon stress with mechanical fixation compared to hand suturing to fixate the mesh. Given the significant reductions in fixation time and the lower workload related stress, it is likely that the ETHICON SECURESTRAP^TM^ Open absorbable strap fixation device might positively influence both demand and resource evaluations and therefore result in a “challenge” state for the surgeon. While future research should explicitly test these predictions, there are potential implications for improved surgical performance, reduced surgeon stress, and decreased medical costs.

Alongside reducing surgeon workload, there are other significant benefits of reducing surgical procedure duration, including reduced rates of complications and decreased hospital length of stay and costs. A study of close to 300,000 operations performed at over 170 hospitals showed that surgical operative duration is associated with increased risk-adjusted infectious complication rates and length of hospital stay [11]. In another study, the duration of surgery was one of the two key predictors of 30-day readmission after open ventral hernia repair [21]. In yet another retrospective analysis of 476 patients with incisional hernia it was found that the operation time is the only significant risk factor associated with mesh graft infection following incisional hernia repair [30]. Other studies have also shown that shorter anesthesia durations may be associated with reduced postoperative nausea and vomiting [31–33] and reduced pulmonary complications [34]. Sinclair et al. found that a 30-minute increase in the duration of anesthesia increased the likelihood of postoperative nausea and vomiting by 59% [33].

While the extent of savings depends on the cost basis, studies have found that small improvements in operating room efficiencies can translate into significant impact on hospital costs. Shìppert estimated $100,000 in savings for only 7-minute reduction in each surgery for 250 cases [29]. In a cost analysis of colectomy, Chatterjee et al. used opportunity cost to demonstrate that the average time of 27 additional minutes of procedure time equates to a missed opportunity cost of $250–$700 [35]. Given that reductions in hospital reimbursements are being linked to unplanned readmissions, there has been an increased effort to accurately track and reduce readmissions [36]. Such efforts include the inclusion of 30-day readmissions in the American College of Surgeons' National Surgical Quality Improvement Program (NSQIP) and analyses of these data to identify predictors of readmission [37, 38].

Our study provides evidence that in open ventral/incisional hernia procedures, compared to IPOM mesh fixation with sutures, mechanical fixation with ETHICON SECURESTRAP^TM^ Open significantly reduces fixation time and lowers surgery workload stress. However, the results need to be viewed in perspective of certain limiting considerations. First, although utmost care was taken to ensure scientific rigor, this study was conducted in a porcine model which might not fully simulate actual human surgical procedure. The application of this mechanical device will need to be further evaluated in human patients and investigated for long term results in terms of efficacy and (lack of) recurrence of ventral hernia. The laparoscopic version of the device has been shown to sustain long term efficacy and low recurrence, results that are expected in the open version as well. Data on the use of the device and its clinical outcomes are planned to be captured in the International Hernia Mesh Registry (IHMR) and should be available for analysis over time. Second, the number of surgeons and procedures were limited and, as such, generalizability of the findings will need to be validated in broader use. Further research is required to elucidate chronic surgeon stress and performance in hernia repair surgeries, and the impact of surgical devices on such. Both the efficacy of surgeon technique and the usability of the fixation device would need to be taken into consideration when evaluating reduction in surgeon stress.

While the fixation method shows ample promise, it may not be suitable for all patients. Surgeon's opinion should be the primary determinant of appropriateness of techniques for individual patients. Future studies will need to further establish the use of the ETHICON SECURESTRAP^TM^ Open device as a new fixation method that is cost-effective and reduces the stress for both the surgeon during the procedure and the patient in terms of avoided recurrences in the long term.

## 5. Conclusion

Stress reduction is a valuable metric by which the potential benefit of new medical devices or techniques is evaluated. In open IPOM mesh repair of ventral/incisional hernias, mechanical fixation with ETHICON SECURESTRAP^TM^ Open demonstrated a significant reduction in fixation time and surgeon stress, which may translate into improved operating efficiency, improved performance, improved surgeon quality of life, and reduced overall costs of the procedure.

## Figures and Tables

**Table 1:** Fixation time difference between suture and mechanical fixation groups.

Paired observation no.	Fixation time in suture group (minutes)	Fixation time in ETHICON SECURESTRAP^TM^ Open group (minutes)	Time difference (minutes)
1	21.00	4.45	16.55
2	30.22	2.57	27.65
3	34.63	5.72	28.92
4	36.63	3.42	33.22
5	43.00	6.93	36.07
6	39.00	5.17	33.83
7	35.72	4.48	31.23
8	30.20	2.77	27.43
9	90.95	3.30	87.65
10	44.37	2.33	42.03
11	44.92	2.37	42.55
12	29.40	2.88	26.52
13	72.40	3.35	69.05
14	42.13	2.88	39.25
15	33.42	5.00	28.42
16	21.95	3.63	18.32
17	37.00	10.47	26.53
18	18.47	5.10	13.37

**Table 2:** Difference in surgery task load between suture and mechanical fixation groups.

Paired observation number	SURG-TLX score in suture group	SURG-TLX score in ETHICON SECURESTRAP^TM^ Open group	Difference in task load
1	65.00	17.00	48.00
2	38.33	5.00	33.33
3	62.33	35.67	26.67
4	70.00	31.67	38.33
5	51.67	29.00	22.67
6	45.67	31.00	14.67
7	41.67	8.33	33.33
8	37.67	15.00	22.67
9	53.67	12.33	41.33
10	34.67	14.67	20.00
11	39.67	16.00	23.67
12	24.33	27.00	−2.67
13	50.00	7.67	42.33
14	13.00	5.00	8.00
15	38.33	30.67	7.67
16	17.00	10.00	7.00
17	28.33	16.33	12.00
18	7.67	7.67	0.00

**Table 3:** Differences in weighted surgical task load dimension scores between suture and mechanical fixation groups.

Paired obs. number	Suture						ETHICON SECURESTRAP^TM^ Open					
	Distractions	Mental demand	Physical demand	Situational stress	Task complexity	Temporal demand	Distractions	Mental demand	Physical demand	Situational stress	Task complexity	Temporal demand
1	30	160	400	220	110	55	10	30	100	60	40	15
2	0	400	110	165	220	40	0	200	80	75	160	20
3	0	350	70	30	220	105	0	125	25	70	140	75
4	20	325	60	30	110	80	20	25	5	5	60	10
5	0	160	260	75	25	285	0	20	40	75	20	30
6	35	150	250	60	100	0	20	45	75	40	60	0
7	60	150	80	260	200	0	15	20	20	20	40	0
8	125	195	150	60	0	45	125	105	60	80	0	90
9	5	40	120	0	140	120	5	40	80	0	60	60
10	15	90	275	80	70	45	5	10	25	20	10	5
11	0	400	160	165	260	65	0	200	60	75	120	20
12	0	325	55	30	200	75	0	200	35	30	140	60
13	20	275	55	15	100	100	20	100	15	10	50	30
14	0	40	80	150	40	210	0	20	60	75	20	45
15	15	75	125	50	100	0	15	60	200	30	100	0
16	15	30	30	40	80	0	15	10	10	20	20	0
17	75	60	30	30	0	60	50	30	20	20	0	30
18	5	10	40	0	20	40	5	10	20	0	40	40

Mean	**23.33**	**179.72**	**130.56**	**81.11**	**110.83**	**73.61**	**16.94**	**69.44**	**51.67**	**39.17**	**60.00**	**29.44**

SD	***33.17***	***133.66***	***102.87***	***77.34***	***80.81***	***74.10***	***29.71***	***69.02***	***46.53***	***29.62***	***50.76***	***27.43***

**Table 4:** Statistical analyses for differences between suture and mechanical fixation groups.

Measure	Number of paired observations	Mean	Median	Std. dev.	Std. error	Lower 95% CL for mean	Upper 95% CL for mean
Time difference in minutes	18	34.92	30.07	17.98	4.24	25.98	43.86

Differences in surgical task load	18	22.17	22.67	15.12	3.56	14.65	29.69
